# Association of serum calcium concentrations with fibrinogen and homocysteine in nondiabetic Korean subjects

**DOI:** 10.1097/MD.0000000000003899

**Published:** 2016-06-17

**Authors:** Hyun Sun Cho, Sung Won Lee, Juyoung Shin, Sung Dae Moon, Je Ho Han, Bong Yun Cha, Eun Sook Kim

**Affiliations:** a Department of Internal Medicine, College of Medicine, The Catholic University of Korea, Seoul, Republic of Korea; b Health Promotion Center, Seoul St. Mary's Hospital, College of Medicine, The Catholic University of Korea, Seoul, Republic of Korea; c Division of Hepatology, Department of Internal Medicine, College of Medicine, The Catholic University of Korea, Seoul, Republic of Korea; d Division of Endocrinology and Metabolism, Department of Internal Medicine, College of Medicine, The Catholic University of Korea, Seoul, Republic of Korea.

**Keywords:** atherosclerosis, calcium, fibrinogen, homocysteine

## Abstract

Considerable evidence shows that increased serum calcium levels are associated with metabolic disorders, cardiovascular disease, and increased mortality. This study investigated whether serum calcium, within a normal range, is significantly associated with serum fibrinogen and homocysteine, markers of increased cardiovascular disease risk in nondiabetic Korean subjects.

A cross-sectional analysis was performed on 1096 subjects (mean age, 55.1 ± 11.1 years; 36.1% women) undergoing a general health checkup. Serum biochemistry was analyzed including serum albumin-corrected calcium (Ca_c_), insulin resistance (IR, using homeostasis model assessment [HOMA]), fibrinogen, and homocysteine.

Compared with patients within the lowest Ca_c_ quartile, those with higher Ca_c_ levels had increased fibrinogen and homocysteine levels as well as an increased proportion of smoking, dyslipidemia, and HOMA-IR. Correlation analyses revealed linear relationships for Ca_c_ with fibrinogen and homocysteine in both genders. After adjustment for confounding factors, serum Ca_c_ was significantly associated with high fibrinogen (odds ratio [OR] for the highest vs the lowest quartile = 1.76, 95% confidence interval [CI] = 1.09–2.83, *P* = 0.02) and homocysteine (OR = 1.83, 95% CI = 1.07–3.11, *P* = 0.027). Multivariate regression models showed that Ca_c_ was linearly associated with fibrinogen (standardized β = 0.14, *P* < 0.001) and homocysteine (standardized β = 0.07, *P* = 0.009).

High normal calcium concentrations were independently associated with increased levels of fibrinogen and homocysteine. Further investigation is needed to validate whether slightly increased calcium levels within the normal range indicate a higher risk of cardiovascular disease.

## 1 Introduction

Calcium is one of the most abundant elements in the body, most of which is sequestrated in bone, while a very small fraction circulates in the blood playing a vital role in numerous biological process.^[1]^ Dynamic increases in intracellular calcium initiates signal transduction whereas cells in a resting state maintain calcium levels 10,000-fold lower than extracellular calcium levels through a complex regulatory system. Serum calcium levels are maintained within a narrow range, by the interplay of various hormones involved in gastrointestinal absorption, intracellular shifts, bone remodeling, and renal excretion. Regarding calcium imbalances, considerable evidence shows that hypercalcemia is associated with increased risks of developing of cardiovascular disease (CVD) and mortality.^[2]^ Recent population-based studies indicate that a slight imbalance in serum calcium, even within a normal range, is closely associated with metabolic disorders^[3–6]^ and predicts development of type 2 diabetes,^[7,8]^ suggesting a physiological link between serum calcium and increased cardiovascular (CV) risk. Although some studies report that high normal serum calcium levels can predict the incidence of myocardial infarction^[9]^ and CV mortality,^[10,11]^ it is still unclear whether slight changes in serum calcium induce CVD. Similarly, the mechanisms linking serum calcium with increased CV events and outcomes remain unclear.

Atherothrombotic CVD is the leading cause of death and disability worldwide. Hemostatic markers are a well-known novel CV risk factors as disturbed homeostasis in coagulation contributes to the development and progression of atherosclerosis.^[12]^ Fibrinogen is one of the most abundant coagulation factor in the blood and increased fibrinogen levels predispose individuals to thrombosis through increased viscosity, inflammation, induction of platelet aggregation and reactivity, formation of dense fibrin clots resistant to fibrinolysis, and promotion of the growth of unstable plaques.^[13]^ Observational studies indicate a positive correlation between fibrinogen concentration and CVD severity,^[12]^ and several prospective studies show that fibrinogen strongly predicts the incidence of coronary artery disease, CV events, and death.^[12]^ Homocysteine is a sulfur-containing amino acid derived from methionine. Raised homocysteine levels are associated with endothelial dysfunction, platelet hyperactivity, and promotion of thrombus formation, all of which increase the risk of CVD.^[14]^

This study investigated whether serum calcium is significantly associated with fibrinogen and homocysteine, important biomarkers of CVD risk, in nondiabetic Korean subjects.

## 2 Methods

### 2.1 Study population

A total of 1096 nondiabetic patients, which was defined as either a fasting serum glucose level equal to or over 126 mg/dL or as the current use of anti-diabetic medication, who visited the Health Promotion Center of Seoul St. Mary's Hospital for general health check-up from April 2009 to March 2011 were enrolled. All patients were aged more than or equal to 20 years and underwent several examinations including blood tests for serum calcium, fibrinogen, and homocysteine. After excluding individuals who had a history of CVD, cerebrovascular disease, or arrhythmia (n = 51) and out of normal range albumin corrected calcium levels (Ca_c_) (n = 10), 1035 subjects were included for the cross-sectional analysis. This study was approved by the institutional review board of Catholic University of Korea, Seoul, Korea.

### 2.2 Measurement of biochemical parameters and definition of variables

All patients completed questionnaires regarding their medical, social, and behavioral history and anthropometric measurements were carried out by standardized methods. Blood samples were collected after overnight fast. Hitachi Modular Chemistry Analyzer (Hitachi, Tokyo, Japan) was used to assess serum calcium and phosphorus by colorimetric method. The normal reference value of serum calcium was 8.0 to 10.0 mg/dL. Sysmex CS5100 coagulation analyzer was used to assess plasma fibrinogen level and the Hitachi 7600 analyzer using an Autolab Homocysteine (IVD Lab Co., Uiwang, Korea) was used to assess the homocysteine level.

Hyperlipidemia was defined as total cholesterol (TC) ≥ 240 mg/dL, low-density lipoprotein cholesterol (LDL-C) ≥ 160 mg/dL, high-density lipoprotein cholesterol (HDL-C) < 40 mg/dL, and triglyceride ≥200 mg/dL or the intake of antidyslipidemia drugs. The National Cholesterol Education Program-Adult Treatment Panel III criteria with the cutoffs for the Asia-Pacific region was used to define metabolic syndrome.^[15]^

### 2.3 Statistical analysis

Analysis of covariance was performed for the comparison of mean variables according to the quartiles of serum corrected albumin calcium (Ca_c_) concentrations. Spearman correlation analyses were performed to examine the association between Ca_c_ levels with various parameters in men and women. Based on the highest quartile of values, high fibrinogen and homocysteine were defined as ≥311.0 mg/dL and ≥12.0 μmol/L, respectively. The risk of high fibrinogen and homocysteine according to Ca_c_ levels was determined by performing logistic regression analysis using the Ca_c_ values acquired after adjustment for age and sex and multivariate adjustment, respectively. To determine the independent association between Ca_c_ concentrations and fibrinogen or homocysteine as a continuous measure, multiple linear regression analysis was performed. *P* values of <0.05 were considered statistically significant. All analyses described above were performed using SAS version 9.1 (SAS Institute, Cary, NC).

## 3 Results

### 3.1 Clinical characteristics according to serum calcium quartile

The mean age of the subjects was 55.1 ± 11.1 years for 639 men and 396 women. The clinical characteristics of subjects grouped according to serum calcium quartile are shown in Table 1. Across serum albumin-corrected calcium (Ca_c_) quartiles, fibrinogen, homocysteine, the percentage of males and current smokers, fasting plasma insulin (FPI), TC, triglycerides (TG), LDL-C, uric acid, and homeostatic model assessment of insulin resistance (HOMA-IR) score increased with calcium levels, whereas age and estimated glomerular filtration rate (eGFR) decreased.

**Table 1:**
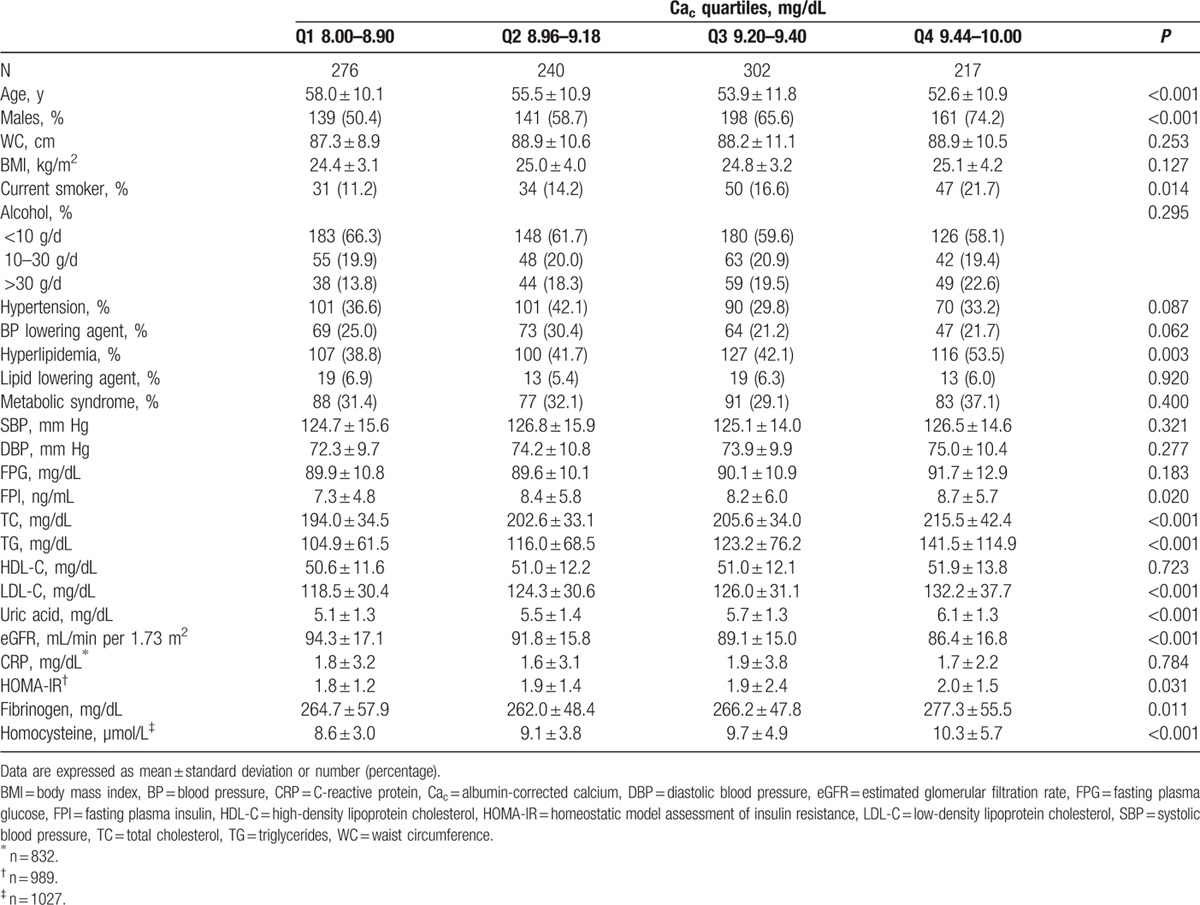
Clinical characteristics of study subjects according to quartiles of serum corrected albumin calcium (Ca_c_).

### 3.2 Association between calcium concentrations and clinical variables according to gender

In age-adjusted partial Spearman correlation analyses, Ca_c_ showed a positive correlation with TC, TG, LDL-C, uric acid, fibrinogen, and homocysteine and a negative correlation with eGFR, in both genders (Table 2).

**Table 2:**
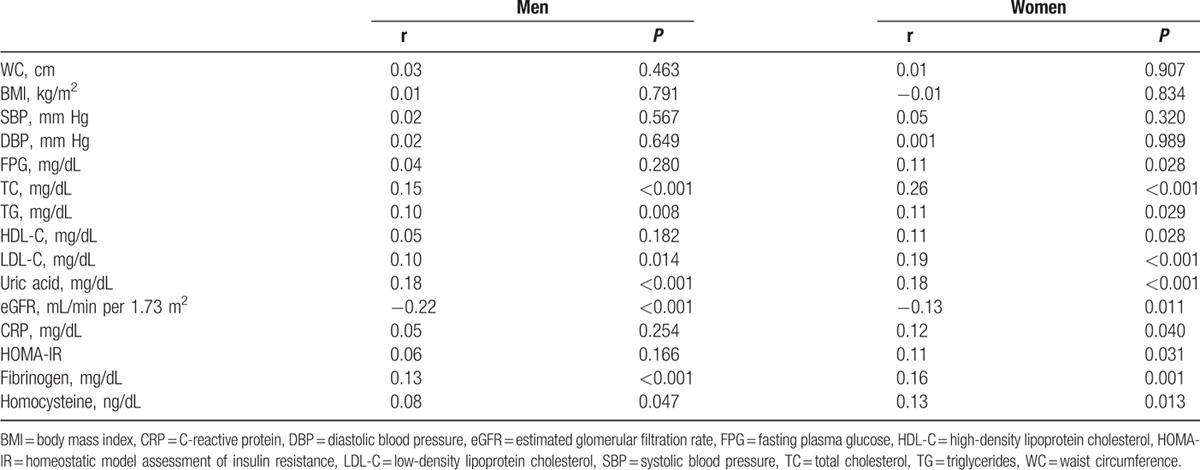
Age-adjusted Spearman correlation between Ca_c_ and clinical variables.

### 3.3 Association between calcium and increased fibrinogen and homocysteine

According to the quartiles of serum Ca_c_, the prevalence of high fibrinogen (18.1%, 16.3%, 17.2%, and 23.5%) and homocysteine increased (11.4%, 13.3%, 15.7%, and 25.7%) with increasing Ca_c_. After adjustment for age, sex, waist circumference, systolic blood pressure, hypertension, hyperlipidemia, current smoking, alcohol consumption, and eGFR, serum Ca_c_ was significantly associated with high fibrinogen (odds ratio [OR] for Q4 vs Q1 = 1.76, 95% confidence interval [CI] = 1.09–2.83, *P* = 0.02) and homocysteine (OR = 1.83, 95% CI = 1.07–3.11, *P* = 0.027) (Table 3).

**Table 3:**
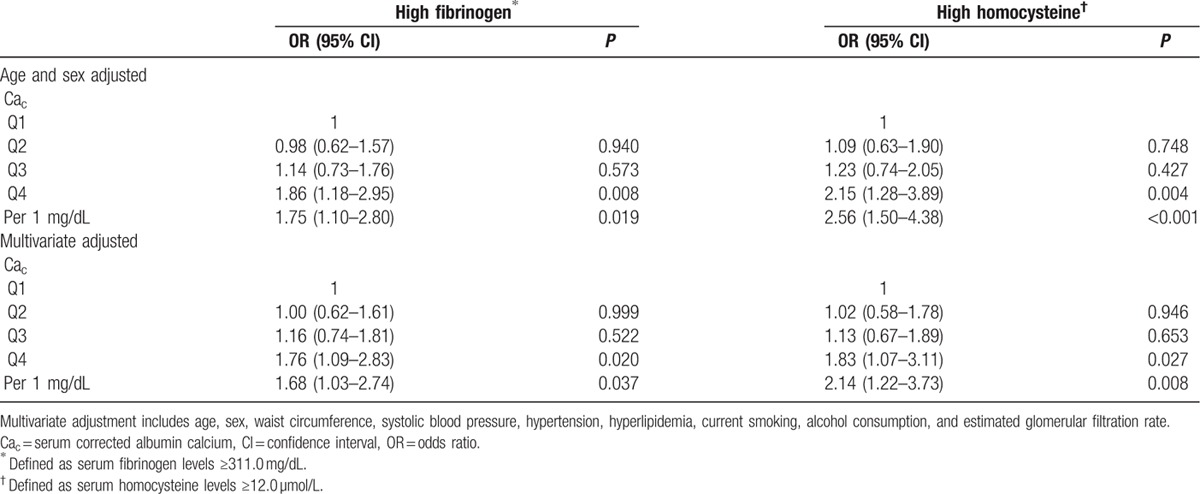
Risk of high fibrinogen and homocysteine according to Ca_c_ levels.

Multivariate regression models were performed to examine the continuous association between Ca_c_ and fibrinogen and homocysteine levels (Table 4). Ca_c_ was linearly associated with fibrinogen (standardized β = 0.14, *P* < 0.001) and homocysteine (standardized β = 0.07, *P* = 0.009) after adjustment for confounding factors.

**Table 4:**
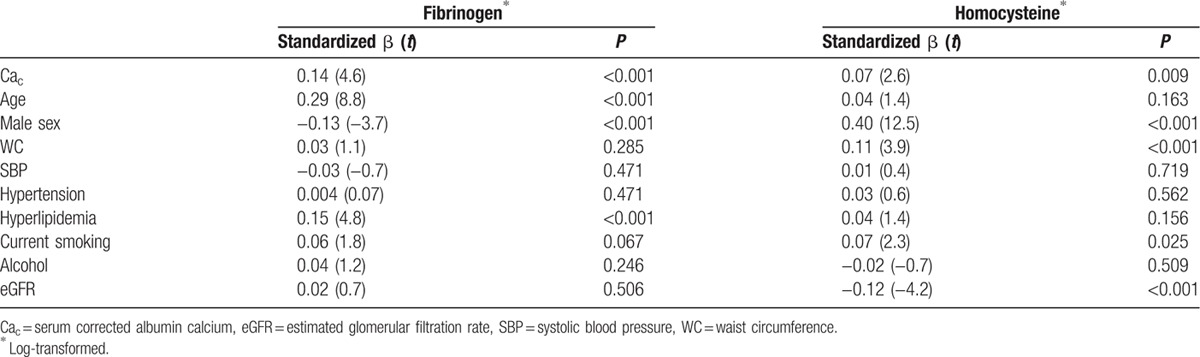
Multivariate linear regression analysis for Ca_c_ and other clinical variables with fibrinogen and homocysteine as the dependent variables.

## 4 Discussion

In this study, we found close association between serum calcium, within the reference range, with fibrinogen and homocysteine in a nondiabetic population. These associations were present in a concentration-dependent manner and remained significant after adjusting for traditional confounding factors. Considering the greater risk of atherosclerosis and thrombosis with increased levels of fibrinogen and homocysteine, our data suggest that slight changes in serum calcium may contribute to CVD, via a pathogenic link to atherothrombosis.

Clinical studies initially noted the increased risk of CVD mortality with excessive calcium levels in chronic kidney disease.^[2]^ The mechanism responsible for increased CVD in hypercalcemia was reported to be associated with vascular calcification either by simple precipitation or osteogenic conversion of vascular smooth muscle cells, along with increased phosphorus levels.^[16]^ A higher incidence of vascular atherothrombogenic events and increased mortality in subjects with increased calcium levels have also been observed in patients with primary hyperparathyroidism, who exhibit hypercalcemia, which is not necessarily accompanied by hyperphosphatemia, suggesting an intrinsic role for calcium per se.^[17]^ Recently, the clinical implications of increased calcium levels in CVD have extended into the normal ranges after findings of functional and structural changes in the vasculature.^[11]^ Previous studies reported that high normal calcium levels are associated with greater pulse wave velocity in 9165 Chinese subjects,^[18]^ carotid plaque thickness in 1194 multiethnic subjects,^[19]^ and increased risk of left ventricular hypertrophy in 833 Chinese diabetic subjects.^[20]^ Recent data from a longitudinal study of 1507 middle-aged Japanese men, which found changes in brachial-ankle pulse wave velocity during a 3-year follow-up period, further highlight the possibility that increased serum calcium may be involved in the pathogenesis of atherothrombosis.^[21]^

The biological mechanisms underlying the association between serum calcium concentrations and increased prothrombotic markers are unclear, but there may be several explanations. First, disturbed calcium homeostasis directly leads to platelet activation, having a key role in the pathogenesis of atherosclerosis and thrombus formation.^[22]^ Calcium is an essential hemostatic cofactor and a ubiquitous second messenger involved in intracellular signaling throughout the entire body including the vascular system. Previously, a few studies showed increased platelet counts and coagulation factors^[23,24]^ as well as increased plasminogen activator inhibitor-1 and decreased thrombin-activatable fibrinolysis inhibitor levels in patients with primary hyperparathyroidism,^[25]^ suggesting that hypercalcemia induces hemostatic imbalance. Experimental hypercalcemia in rats results in a reduced clotting time, providing a physiological background for the clinical data.^[26]^ Second, serum calcium levels may mediate atherothrombosis by its close association with dyslipidemia, an established CV risk factor. Previous data demonstrate that serum calcium levels are closely associated with metabolic disturbances including dyslipidemia, hypertension, abnormal glucose metabolism, and metabolic syndrome.^[3,5]^ Accordingly, we also found significant correlations between serum calcium and TG and LDL-C. However, there were no correlations between serum calcium and glucose or blood pressure profiles, probably due to the study population, which included only nondiabetic subjects. LDL has been reported to not only contribute to the pathogenesis of atherosclerosis but also to increase platelet activity and aggregability.^[27]^ Subjects with increased LDL levels also have higher β-thromboglobulin, soluble CD40 ligand, and platelet activation marker levels. Furthermore, in vitro, platelets show hyperaggregabilty as well as increased fibrinogen binding, P-selectin expression, and thromboxane A2 (TXA2) production.^[27]^

In addition, the association between serum calcium and HOMA-IR scores could provide a further explanation of the thrombotic potential observed with increased serum calcium levels. Insulin ameliorates platelet aggregation by nitric oxide synthase induction, nitric oxide production, and vasodilation.^[28]^ Thus high serum calcium levels may predispose the hemostatic system to activation as a result of the loss of antithrombotic action following insulin resistance.^[29]^ Consistent with our data, elevated serum calcium levels are associated with insulin resistance in both primary hyperparathyroidism^[30]^ and healthy subjects.^[8]^ Moreover, several recent studies show that serum calcium levels predict the incidence of type 2 diabetes.^[7,8]^ Experimental data demonstrate that increased cytosolic calcium levels attenuate the effect of insulin on glucose uptake by decreasing insulin receptor activity and reducing glucose transporter expression in adipocytes and muscle cells.^[31,32]^

Lastly, calcium may mediate hemostatic imbalance through increased oxidative stress, which has been implicated as a common pathway promoting atherosclerosis and thrombosis.^[33]^ Excessive calcium may interfere with mitochondrial β-oxidation and increase reactive oxygen species (ROS) production, facilitating lipid oxidation, platelet activation, and endothelial dysfunction.^[34]^ Reciprocally, oxidative stress from hyperlipidemia and insulin resistance could contribute to calcium dysregulation and hemostatic imbalance in a complex manner, resulting in a significant link between serum calcium and proatherogenic status.^[35]^

### 4.1 Clinical implications of the study

In cases of hypercalcemia, many studies report the beneficial effects of lowering calcium levels. However, for subjects with normal calcium levels, the beneficial effects of manipulating serum calcium are doubtful.^[36]^ However, serum calcium levels may reflect increased thrombotic potential through oxidative stress or insulin resistance. Alternatively, shared precedent factors such as genetic or environmental determinants may underlie the association between serum calcium and increased prothrombic markers. As about 70% of the variation in total calcium levels is attributed to genetic influence, variation in genes affecting calcium levels may be involved in coronary heart disease and CV mortality. Although a previous study suggested a single nucleotide polymorphism (SNP) of *CASR* (rs1801725) as a predictor of CV mortality in 2561 Europeans with coronary artery disease, no association between variants of the *CASR* locus was observed in a genome-wide association meta-analysis of 39,400 individuals.^[37]^ Further studies are needed to determine the genetic influence of other potential genes such as *CSTA*, *DGKD*, *GCKR*, and other variants of *CASR*.^[38]^ The results of the present study have important clinical implications. In contrast to parathyroid hormone (PTH) or vitamin D, serum calcium levels are easy to measure and cost effective which makes it a potential screening marker for high CV risk.

### 4.2 Limitations of the study

This study has several limitations. First, the cross-sectional design of our study was not sufficient to demonstrate a causal relationship among serum calcium, fibrinogen, and homocysteine. Second, we could not evaluate the influence of PTH and vitamin D levels on the relationship between calcium and atherothrombosis. Although the effects of PTH and vitamin D may have been mitigated as we analyzed the subjects with normal calcium levels, we still cannot preclude the possibility of secondary hyperthyroidism caused by low serum 25-hydroxyvitamin D levels which is prevalent in Korean subjects^[39]^ and its influence on CVD considering the close association between PTH and CVD.^[40]^ Third, we have no information on the dietary intake of calcium or vitamin D or medications, factors that could affect serum calcium, fibrinogen, and homocysteine levels. Although previous studies report the beneficial impact of calcium supplements on CVD events and mortality, this remains controversial because of compelling contradictory results.^[41]^ Moreover, several studies’ findings of the lack of correlation between calcium supplements and serum calcium levels suggest that the intake of calcium supplements (administered either via food or medication) may not always influence serum calcium levels.^[42]^ In addition, no information on medication was available, which may have biased the observed association. Fourth, we used single values of serum markers, which are less accurate than using the means of several measures, and we did not measure ionized calcium levels, which is the gold standard. However, the use of standardized methods set in a single center and obtaining serum measurements in a fasted state likely improved reliability by reducing the influence of diet on biochemical markers.

## 5 Conclusions

Serum calcium levels are significantly associated with fibrinogen and homocysteine levels, in a concentration-dependent manner, in nondiabetic Korean subjects. Further longitudinal studies and biological mechanistic investigations are needed to verify whether calcium levels indicate the risk of CVD and further elucidate the pathogenic role of calcium in atherothrombosis.
